# An Analysis of the Impact of Intra-abdominal Pressure on Surgical Outcomes in Cases of Intestinal Obstruction: A Prospective Observational Study

**DOI:** 10.7759/cureus.59736

**Published:** 2024-05-06

**Authors:** Siva Sumanth Dukkipati, Ashok K Puranik, Satya Prakash Meena, Mayank Badkur, Mahendra Lodha, Prathyusha V Kompally, Ramkaran Chaudhary, Mahaveer Singh Rodha, Naveen Sharma

**Affiliations:** 1 General Surgery, All India Institute of Medical Sciences, Jodhpur, Jodhpur, IND

**Keywords:** abdominal compartment syndrome, intra-abdominal pressure, intestinal obstruction, intra-abdominal hypertension, hospital stay, fascial dehiscence, bowel ischemia

## Abstract

Background: The decision and timing of surgical exploration of intestinal obstruction depend on the clinical findings and probable etiology of the symptoms. Patients with intestinal obstruction often have intra-abdominal hypertension (IAH), which is associated with a poor prognosis.

Purpose of the study: The purpose of the study is to evaluate the surgical outcomes in patients with intestinal obstruction in relation to intra-abdominal pressure (IAP).

Materials and methods: The study was conducted on 50 patients with intestinal obstruction undergoing surgery. Preoperatively, IAP was measured in all the patients and was allocated into two groups based on the presence or absence of IAP. Patients were assessed for the postoperative length of hospital or ICU stay, surgical site infection, wound dehiscence, and recovery following surgery.

Results: The patients with preoperative IAH had significantly longer postoperative stays, with a median stay of eight days in these patients compared to four days in patients without IAH (p=0.009). A significantly higher number of patients (24%) had gangrenous changes on the bowel wall (p=0.042) and fascial dehiscence (p=0.018) in the group associated with raised IAP. A total of 75% of patients who required ventilator support belonged to the raised IAP group. The mean IAP in patients admitted to the ICU was significantly higher than in patients not admitted to the ICU (p=0.027).

Conclusion: Preoperative IAH in intestinal obstruction is a significant factor in predicting the possibility of bowel ischemia with gangrene, perforation, intra-abdominal sepsis, surgical site infections, and prolonged hospital stay. Early surgical exploration and abdominal decompression must be considered in such cases.

## Introduction

Intra-abdominal hypertension (IAH) is characterized by a sustained or pathological increase in intra-abdominal pressure (IAP) of ≥12 mm Hg (16.3 cm of H2O). Abdominal compartment syndrome (ACS) is defined as a sustained IAP exceeding 20 mmHg, accompanied by organ dysfunction [1]. Various conditions can lead to elevated IAP, such as intestinal obstruction, peritonitis, ascites, intra-abdominal hemorrhage, intra-abdominal tumors, abdominal and pelvic trauma, laparoscopic surgery, and peritoneal dialysis [2]. These increased pressures can result in compromised pulmonary and renal function, oliguria, reduced cardiac output, cerebral ischemia, intestinal edema, and bowel ischemia [3-7].

## Materials and methods

Study design, setting, size, and duration

A prospective observational study was conducted at a tertiary healthcare center in North India over a period of 20 months, from February 2019 to December 2020. The study included a sample size of 50 patients.

Participants

The study included all patients over the age of 18 with a diagnosis of intestinal obstruction who were undergoing surgery. Pregnant patients and those with post-traumatic obstruction were excluded from the study.

Aim of the study

The study aimed to assess the surgical outcomes in patients with intestinal obstruction in relation to IAP.

Sample size calculation

The sample size was calculated from median values in reference to Murtaza et al.'s study [8], considering the mean length of stay in patients with high IAP as 5.25 (SD 2.0361) and patients without high IAP as 3.25 (SD 0.9014). We estimated a sample size of 17 per group at a 95% confidence interval and 95% power. After considering a further contingency of 40%, the sample size required was 25 per group.

A detailed history, general and abdominal examination, all general routine blood investigations, X-ray abdomen (erect and supine), and CECT of the whole abdomen were performed. The IAP of each patient was measured at the time of presentation and before the placement of a nasogastric tube. The patients were divided into two groups based on their IAP: Group A had IAH, and Group B did not have IAH. According to IAP, the first 25 patients were included in each group.

Intra-abdominal pressure measurement procedure

**Figure 1:**
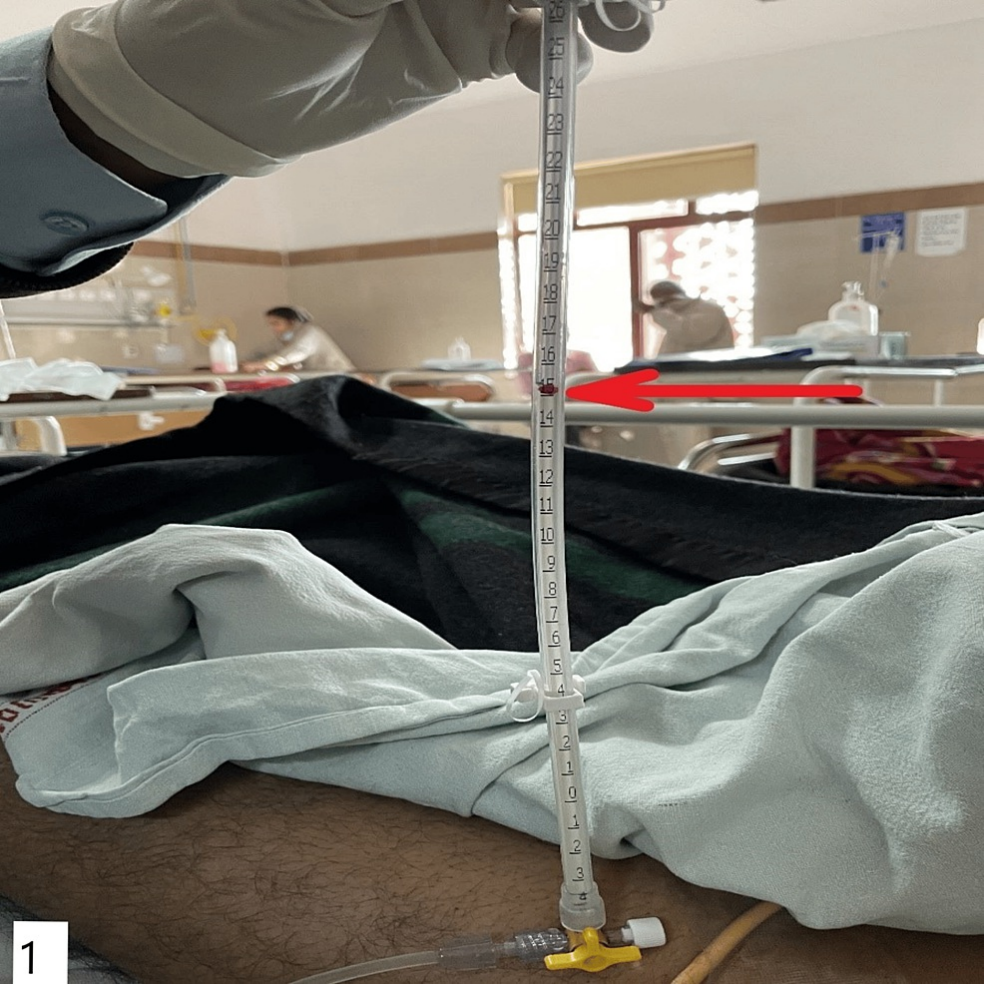
Manometer placement and position of the patient to measure IAP IAP: intra-abdominal pressure

The individuals were catheterized using a three-way Foley catheter, and their IAP was monitored through their irrigation limb, which was linked to a manometer. A urobag was attached to the drainage limb. Following the clamping of the drainage tube, 50 mL of normal saline was introduced into the bladder via the catheter. The irrigation limb, connected to a manometer, was utilized to gauge the flow of the injected normal saline. During the end-expiration phase, the patient was placed supine, and the manometer was positioned at the level of the symphysis pubis (Figure 1) [9].

The IAP value was determined by the level to which the fluid ascended in the manometer. IAP measurements were taken upon admission and prior to the decision for surgery. The highest value was considered in the analysis.

All patients underwent a standard preoperative assessment. Postoperatively, patients were monitored for the length of their hospital or ICU stay, as well as for any surgical site infections, wound dehiscence, and recovery progress after the surgery. The IAP was linked to both mortality and morbidity. Ultimately, outcomes for both groups were documented and compared.

Statistical analysis

Data was collected in the paper-based case record form prior to, during, and after operations. Subsequently, this data was transferred to Microsoft Office Excel (Microsoft Corporation, Washington, USA). The statistical analysis was conducted by a statistician who had no involvement in patient care. For the statistical analysis, SPSS Statistics version 23.0 (IBM SPSS for Windows version 23.0, 2015, Armonk, NY: IBM Corp.) was utilized. The mean, median, and interquartile range (IQR) were used to describe the ordinal data, while the Mann-Whitney U test was employed for comparison. Frequency and percentage were used to report the nominal data, and the Chi-Square test was used for comparison. Statistical significance was indicated by two-sided p-values of less than 0.05.

Ethical considerations

Ethical clearance was obtained from the Institutional Ethics Committee of the All India Institute of Medical Sciences, Jodhpur (AIIMS/IEC/2018/786). Patients were enrolled after giving informed consent.

## Results

The study involved 50 patients who were divided into two groups: Group A consisted of patients with an IAP of less than 12 mm Hg or 16.3 cm H2O, while Group B included patients with an IAP greater than 12 mm Hg or 16.3 cm H2O. The average age of patients in Group A was 49.8 years, while in Group B, it was 46.3 years. The age distribution was similar in both groups. Both groups had 12 women and 13 men, making the sex distribution comparable as well. The mean IAP in Group A was 13.7 cm H2O, while in Group B, it was 17.9 cm H2O. The overall mean IAP was 15.8 cm H2O, with the highest recorded IAP being 22.4 cm H2O and the lowest being 10.5 cm H2O. The markers of septicemia, such as leukocyte counts and serum creatinine levels, were similar in both groups. Group A had a higher proportion of patients with small bowel obstruction compared to Group B (p=0.077). The median length of hospital stay was significantly longer in Group B compared to Group A (p=0.009) (Table 1).

**Figure 2:**
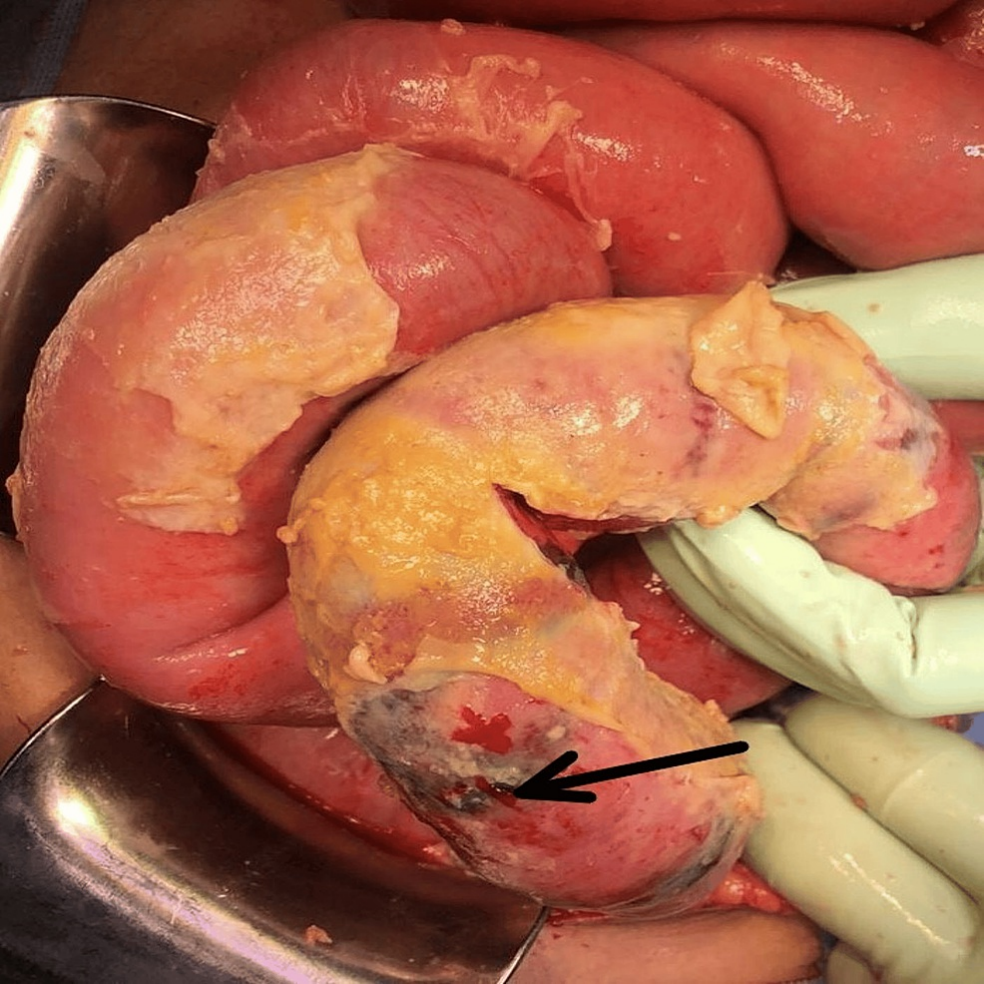
Gangrenous changes in the bowel wall

The patients in both groups had various causes of intestinal obstruction, including adhesions or strictures (almost half of the total, 50%), malignancy (32%), hernias (10%), volvulus (4%), and intussusception (2%). Group B had a significantly higher number of cases with gangrenous changes in the bowel wall compared to Group A (p=0.042) (Figure 2).

**Table 1:** Demographic criteria of Group A and Group B Data is presented as n (%), and an asterisk (*) shows a significant p-value

Demography		Group A (n=25) (50%)	Group B (n=25) (50%)	P-value
Age (years) (mean+/-SD)		49.8 ± 19.9	46.3 ± 16	
Level of obstruction	Small bowel	19 (76%)	13 (52%)	0.077
	Large bowel	6 (24%)	12 (48%)	
Leucocyte count (x10^9^/L)		10.6 ± 4.2	12.1 ± 7.7	0.946
Creatinine (mg/dL)		1.0 ± 0.4	1.1 ± 0.7	0.846
Postoperative stay (no. of days) (median) (Q1-Q3)		4.0 (4.0-6.0)	8.0 (5.0-11.0)	0.009*

There was no statistically significant difference between the groups in terms of surgical site infections, although Group B had a higher number of patients with such infections (p=0.157). Group B also had a significantly higher number of cases with fascial dehiscence in the postoperative period (p=0.018), while none of the patients in Group A experienced fascial dehiscence. In patients who were admitted to the ICU following surgery, 75% belonged to Group B, which was higher but not statistically significant (p=0.609). The mortality rate was the same in both groups, with two cases in each group (p=1.0) (Table 2).

**Table 2:** Perioperative outcome of the study patients Data is presented as n (%), and an asterisk (*) shows a significant p-value ICU: intensive care unit

Postoperative outcomes	Group A (n=25) (50%)	Group B (n=25) (50%)	P-value
Gangrenous bowel changes	1 (4%)	6 (24%)	0.042*
Surgical site infections	3 (12%)	7 (28%)	0.157
Fascial dehiscence	0 (0%)	5 (20%)	0.018*
ICU admission	1 (4%)	3 (12%)	0.609
Mortality	2 (8%)	2 (8%)	1.0

The average postoperative stay in the hospital was nearly double that of patients with surgical site infections and was found to be statistically significant (p=0.001). The mean IAP observed in patients requiring ICU admission was 18.7 cm H2O, which was significantly higher (p=0.027). It was noted that mortality was higher in patients admitted to the ICU (75%) and was found to be statistically significant (p=0.001) (Table 3).

**Table 3:** Hospital stay and mortality of patients following surgical site infections and ICU admissions An asterisk (*) shows a significant p-value ICU: intensive care unit, IAP: intra-abdominal pressure

Parameter	Yes	No	P-value
Surgical site infection			
Postoperative stay (no. of days) (mean ± SD)	10.5 ± 3.30	6.13 ± 3.52	0.001*
ICU admission			
IAP (cm H2O) (mean ± SD)	18.7 ± 3.61	15.57 ± 2.53	0.027*
Mortality (n)	3	1	0.001*

## Discussion

The research findings indicated that the primary reasons for obstruction were inter-bowel adhesions, bands, and strictures, with malignancy affecting the large colon and rectum following closely. The prevalence of adhesions and hernias as causes of obstruction aligns with existing literature [10]. However, the study revealed a higher occurrence of malignancy leading to obstruction compared to hernia, attributing this discrepancy to the inclusion of solely operated patients in the analysis. In the context of measuring IAP, various methods such as intracolonic, intragastric, intravesical (bladder), or inferior vena cava catheters can be employed [11]. A practical approach to screening for IAH and ACS involves monitoring bladder pressure (intravesical pressure), a technique that can be easily implemented in peripheral healthcare facilities [9,12]. The investigation noted that IAP exhibited variability based on the underlying causes. Interestingly, there was no significant distinction in mean values of blood parameters like leucocyte count, serum electrolytes, urea, and creatinine levels between the different groups, contrasting with prior studies that reported abnormalities in blood parameters among individuals with IAH [13].

ACS is a condition characterized by sustained and elevated IAP, which can lead to multiorgan dysfunction syndrome, organ failure, and ultimately death in critically ill patients [14]. The primary approach to managing ACS is to relieve the pressure by decompressing the abdomen through various methods such as supportive care, nasogastric tube placement, paracentesis, percutaneous catheter drainage, or surgery. Surgical decompression is considered the definitive treatment. If at least 1 liter of fluid cannot be drained and the IAP cannot be reduced by at least 9 mmHg within the first four hours after decompression, it is considered a failure, and urgent open abdominal decompression is necessary [12,15]. Treatment for patients with an IAP between 15 and 25 cm H2O should be based on their clinical condition, but it may be difficult to justify without renal or pulmonary compromise. Most patients with an IAP between 25 and 35 cm H2O will require decompression [16]. In addition to causing irreversible damage to the mitochondria and necrosis of the gut mucosa, IAH may also decrease MBF in the intestinal mucosa, increase intestinal permeability, and cause endotoxemia, according to Cheng et al.'s study [17]. A similar association between ACS and bowel ischemia was also observed in patients with severe pancreatitis [18].

A significant difference in the length of hospital stay following surgery was observed between the two groups in this study. Patients in Group B had a median length of hospital stay that was twice as long as those in Group A. This difference can be attributed to the higher incidence of surgical site infections and the longer time required for the bowel to return to its normal activity in patients with higher pressures at the time of presentation. Previous studies conducted by Iyer et al. and Kyoung et al. also found that patients with IAH tended to have longer hospital stays [19,20]. Surgical site infections are a major cause of morbidity and prolonged hospital stays in surgical patients. The higher incidence of surgical site infections in Group B can be explained by the increased pressures in the bowel, which can lead to ischemic and gangrenous changes in the bowel wall and perforation. This perforation can cause intra-abdominal sepsis, hindering wound healing and increasing the risk of surgical site infections. Another study by Singla et al. found that higher IAP scores increase the risk of developing sepsis in the abdomen [21]. Similarly, a study by Agrawal et al. observed an increase in the incidence of surgical site infections and wound dehiscence in cases with increased IAP [22].

The majority of surgical site infections were found in cases of small bowel obstruction, accounting for 80% of the cases. Fascial dehiscence, on the other hand, was only observed in Group B. Interestingly, all patients with fascial dehiscence had previously experienced a wound site infection before the dehiscence occurred. The study revealed that 75% of patients requiring ICU care belonged to Group B, which had IAH. This suggests that patients with higher abdominal pressures may require ventilator support after surgery compared to those with lower pressures. Among the patients admitted to the ICU, one individual had to undergo re-exploration surgery due to gangrenous changes in the bowel and also experienced a surgical site infection during the postoperative period. Unfortunately, this patient passed away on the eleventh day following the initial surgery due to septic shock. The study also found that there was an increased incidence of ICU admission in patients with elevated pressures, which could lead to pulmonary compromise and extrinsic compression of the lungs by the elevated diaphragm. In fact, three out of four patients (75%) who required ventilator support after surgery ultimately died in the study. Similar findings were reported by Iyer et al. and Kyoung et al., who both observed longer ICU stays and higher mortality rates in patients with elevated IAH [19,20].

The research findings indicated that 8% of the total patients included in the study experienced mortality. Among the patients in Group B, which had IAH, two patients passed away. One of these patients had a history of peptic perforation repair and required resection of the diseased bowel due to multiple inter-bowel adhesions and gangrenous patches on the bowel wall. Despite receiving ventilator support, this patient, unfortunately, succumbed on the 11th day post-surgery with an IAP of 17.6 cm H2O.

The second patient in Group B who did not survive had rectal malignancy and suffered from a closed-loop obstruction at the distal ileum with gangrenous changes and loop perforation. This patient had a higher IAP of 22.1 cm H2O and developed multiple organ dysfunction syndrome (MODS) due to renal and pulmonary function derangement, leading to death on the fourth day after surgery. Although the statistical correlation was not significant (p=1.0), it was evident that mortality in patients with IAH (Group B) was linked to MODS, as supported by studies conducted by Khan et al., Agrawal et al., and Beltrán et al., which highlighted the increased risk of organ failure with elevated IAP values [13,22,23].

In order to enhance the analysis and yield more significant results, it would have been beneficial to incorporate conservatively managed patients who did not undergo surgery as part of this study. This limitation should be acknowledged, as it could have provided valuable insights. Additionally, it is important to note that the study did not assess comorbidities such as cardiac and respiratory diseases, which can potentially confound the outcomes and contribute to poorer patient outcomes during their hospital stay.

## Conclusions

Preoperative IAP is found to be a significant predictor of both hospital stay and patient morbidity. To predict IAP, measuring intravesical pressure is an easy, feasible, and effective method. The study reveals a clear association between preoperative IAH and various complications such as bowel ischemia, gangrene or perforation, intra-abdominal sepsis, surgical site infections, and prolonged hospital stay. In cases where patients have a higher IAP, early surgical exploration and abdominal decompression should be considered.
